# River flow prediction in data scarce regions: soil moisture integrated satellite rainfall products outperform rain gauge observations in West Africa

**DOI:** 10.1038/s41598-020-69343-x

**Published:** 2020-07-27

**Authors:** Luca Brocca, Christian Massari, Thierry Pellarin, Paolo Filippucci, Luca Ciabatta, Stefania Camici, Yann H. Kerr, Diego Fernández-Prieto

**Affiliations:** 1 Research Institute for Geo-Hydrological Protection, National Research Council, Via della Madonna Alta 126, 06128 Perugia, Italy; 2 University Grenoble Alpes, CNRS, IRD, Grenoble INP, IGE, 38000 Grenoble, France; 3 Centre d’Etudes Spatiales de la BIOsphère, Université Toulouse 3 CNES CNRS IRD, INRA, Toulouse, France; 4 European Space Agency (ESA), Frascati, Italy

**Keywords:** Hydrology, Natural hazards

## Abstract

Satellite precipitation products have been largely improved in the recent years particularly with the launch of the global precipitation measurement (GPM) core satellite. Moreover, the development of techniques for exploiting the information provided by satellite soil moisture to complement/enhance precipitation products have improved the accuracy of accumulated rainfall estimates over land. Such satellite enhanced precipitation products, available with a short latency (< 1 day), represent an important and new source of information for river flow prediction and water resources management, particularly in developing countries in which ground observations are scarcely available and the access to such data is not always ensured. In this study, three recently developed rainfall products obtained from the integration of GPM rainfall and satellite soil moisture products have been used; namely GPM+SM2RAIN, PRISM-SMOS, and PRISM-SMAP. The prediction of observed daily river discharge at 10 basins located in Europe (4), West Africa (3) and South Africa (3) is carried out. For comparison, we have also considered three rainfall products based on: (1) GPM only, i.e., the Early Run version of the Integrated Multi-Satellite Retrievals for GPM (GPM-ER), (2) rain gauges, i.e., the Global Precipitation Climatology Centre, and (3) the latest European Centre for Medium-Range Weather Forecasts reanalysis, ERA5. Three different conceptual and lumped rainfall-runoff models are employed to obtain robust and reliable results over the 3-year data period 2015–2017. Results indicate that, particularly over scarcely gauged areas (West Africa), the integrated products outperform both ground- and reanalysis-based rainfall estimates. For all basins, the GPM+SM2RAIN product is performing the best among the short latency products with mean Kling–Gupta Efficiency (KGE) equal to 0.87, and significantly better than GPM-ER (mean KGE = 0.77). The integrated products are found to reproduce particularly well the high flows. These results highlight the strong need to disseminate such integrated satellite rainfall products for hydrological (and agricultural) applications in poorly gauged areas such as Africa and South America.

## Introduction

Rainfall is the most important and challenging variable to be measured to obtain accurate river flow predictions, i.e., for river discharge simulation through rainfall-runoff modelling^1–4^. The availability of accurate and near real-time rainfall observations is critical and in many regions of the world ground observations are not available or not accessible^5,6^. Specifically, in many regions of Africa and South America the density of ground networks is approximately one station every 100,000 km^2^ (or less) that is clearly insufficient to obtain reliable river flow predictions^7,8^. In addition to rain gauges, and ground meteorological radars, meteorological and numerical weather prediction models and satellite observations can be used. However, both data sources rely directly or indirectly on ground observations (e.g., through assimilation for modelling or used as reference for satellite products), and hence their reliability over scarcely gauged areas is highly uncertain^9^.

In the recent years, satellite rainfall products have been largely improved, mainly thanks to the successful launch of the global precipitation measurement (GPM) core satellite in 2014^10^. GPM provides quasi-global rainfall measurements by integrating a constellation of geostationary and polar-orbiting satellite sensors through a “top down” approach, i.e., based on the inversion of the upwelling radiation or backscattered signal (for radars) by atmospheric hydrometeors that is related to the surface precipitation rate. GPM is currently providing three operational products based on the Integrated Multi-Satellite Retrievals for GPM (IMERG) algorithm, i.e., Early, Late and Final Run. The three versions have spatial and temporal resolution of 0.1-° and 30-min and different latency: 4 h, 12 h and 3.5 months for the Early, Late, and Final Run, respectively^11^.

In parallel to these developments, in the last 5 years, new “bottom up” approaches based on the inversion of the satellite soil moisture signal have been developed that provide accumulated rainfall estimates between two satellite overpasses^12,13^ or the correction/enhancement of top down products based on soil moisture signal (e.g.,^14,15^). The bottom up approach clearly distinguishes from the state-of-the-art top down method (i.e., GPM) as the rainfall signal is obtained\enhanced from the knowledge of the soil moisture signal, i.e., from the bottom up. This relatively new approach has been tested with different satellite sensors (e.g., Soil Moisture Ocean Salinity, SMOS, mission^15,16^; Soil Moisture Active and Passive, SMAP, mission^17^; and Advanced SCATterometer, ASCAT, sensor^18^), and recently also by integrating multiple satellite soil moisture products^9,19^.

Top down and bottom up approaches have their own advantages and limitations. Several studies have demonstrated clearly that their integration is providing a higher quality rainfall product^9,14–16,20,21^. For hydrological applications such as river flow prediction, the bottom up approach has been found to perform well^22,23^, and the integration with the top down approaches particularly useful^1,24–26^. For instance, Camici et al.^1^ have tested different satellite precipitation products for river flow prediction over 15 catchments in the Mediterranean Basin and have demonstrated that integrating top down and bottom up approaches improves the simulation of discharge for all basins.

In data scarce regions, the assessment of the quality of satellite precipitation is limited from the availability of ground observations and alternative approaches need to be implemented^27^. Recently, Massari et al.^6^ proposed the Triple Collocation approach^28^ for the assessment of satellite rainfall products in ungauged areas by profiting from three independent datasets. Massari et al.^9^ and Brocca et al.^18^ have recently performed Triple Collocation for assessing newly derived satellite rainfall products obtaining that the products based on the bottom up approach^18^, and on the integration of bottom up and top down approaches^9^, have very good performance in Africa and South America. Based on these results, we believe it is important and urgent to carry out the hydrological validation of such products in Africa, where there is a strong need for accurate rainfall data. Due to the scarcity of stream gauges, the hydrological validation of satellite rainfall products in Africa has been carried out on a limited number of studies as recently reviewed by Maggioni and Massari^4^. Previous studies (e.g.,^1,2,29–32^) evaluated the performance of classical state-of-the-art satellite rainfall products such as CMORPH (Climate Prediction Center Morphing technique^33^), TMPA (Tropical Rainfall Measuring Mission Multi-Satellite Precipitation Analysis^34^), and PERSIANN (Precipitation Estimation from Remotely Sensed Information using Artificial Neural Networks^35^). All these studies pointed out that after bias correction of satellite precipitation products, and in some studies also after model recalibration, improved performances in terms of river flow simulation were obtained with respect to the use of original products. However, to our knowledge, the hydrological validation of GPM IMERG products in Africa has not been yet carried out.

On this basis, the main purpose of this study is to investigate the accuracy of top down and bottom up satellite rainfall products for hydrological prediction in data scarce regions. Specifically, we have selected 10 basins in Europe (4), West Africa (3) and South Africa (3) for which daily stream gauge observations in the period 2015–2017 are available, i.e., after the launch of GPM core satellite. The three regions are characterized by good (Europe), medium (South Africa) and poor (West Africa) density of rain gauges. Our main focus is the understanding of the capability of satellite rainfall products in data scarce regions, therefore we have targeted African basins. Four satellite rainfall products have been assessed: (1) IMERG Early Run, (2) GPM+SM2RAIN (^9^, available here: https://doi.org/10.5281/zenodo.3345322), (3) precipitation Inferred from Soil Moisture (PRISM) applied to SMOS, i.e., PRISM-SMOS, and (4) PRISM-SMAP. The two last PRISM-based products have been obtained from the integration of IMERG Early Run with SMOS and SMAP soil moisture through the procedure described in^15,36^. Even though not useful for our target application, for completeness, the 5th European Centre for Medium-Range Weather Forecasts (ECMWF) reanalysis (ERA5^37^), the ground-based Global Precipitation Climatology Centre product (GPCC^38^) and the gauge-corrected IMERG Final Run product (GPM-FR)^11^ have been considered. We underline, however, that the latter 3 products use directly (GPCC and GPM-FR) and indirectly (ERA5) ground observations and hence they are available only with 1+ months latency. Therefore, their use for operational river flow prediction or water resources management in the real-world is not feasible and are used here just for comparison. Moreover, in developing countries, the accessibility to ground observations is not always ensured. To obtain robust results, three different rainfall-runoff models have been used: MISDc (Modello Idrologico Semi-Distribuito in continuo^44^), GR4J (modèle du Génie Rural à 4 paramètres Journalier^45^), and HYMOD (HYdrologic MODel^46^).

The two main research questions addressed in this study are:

1. Do the latest satellite rainfall products outperform ground-based datasets? Are such products useful for river flow prediction in scarcely gauged regions such as Africa?
2. Is there a benefit in the integration of top down and bottom up approaches for river flow prediction?

## Results

The assessment of the satellite products for river flow prediction in the investigated basins has been performed in different steps. Firstly, the three rainfall-runoff models have been applied to all basins to identify the more suitable model for performing the analysis. Secondly, thanks to the high density of rain gauge stations in Europe, a detailed analysis is carried out in which both rainfall and hydrological validation has been performed. Thirdly, triple Collocation has been used to investigate the quality of the products in Africa in terms of rainfall. Finally, the products performance in terms of river flow prediction in Africa has been assessed.

### Selection of the more suitable rainfall-runoff model

**Figure 1:**
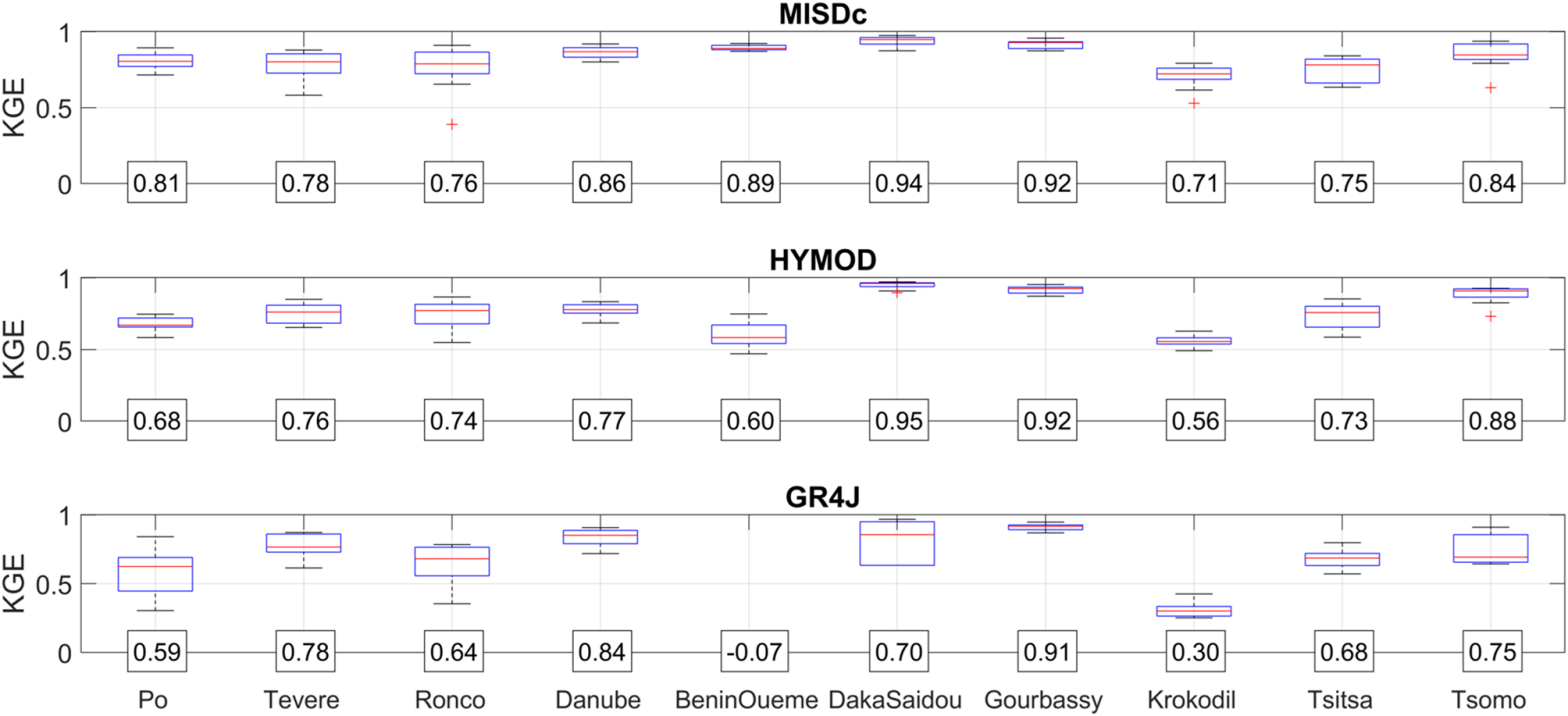
Kling–Gupta Efficiency in the different basins for all products and for the three rainfall-runoff models. For each boxplot, the horizontal line represents the median values and the box represents the 25th and 75th percentile, the dotted whiskers extend to the extreme data points and cross symbols represent outliers. The numbers in the boxes represent the mean values.

As described in methods, the three rainfall-runoff models have been calibrated for the whole period of observations for each basin and rainfall product. The three models have been run in the lumped-mode, i.e., by considering the input rainfall and temperature data spatially aggregate at the basin scale. To investigate the variability of model performance in the different basins, Fig. 1 shows as boxplots the results in terms of Kling–Gupta efficiency (KGE^39^) for all products, separated by basin and by models. For each boxplot, the horizontal line represents the median values and the box represents the 25th and 75th percentile, the dotted whiskers extend to the extreme data points and cross symbols represent outliers. As it can be seen, mean performances for the different basins should be considered satisfactory for MISDc and HYMOD models, i.e., KGE greater than 0.71 and 0.56. For many basins, mean KGE is greater than 0.80 indicating that at least for some models and products the performance in reproducing river discharge is very good. Some models or input rainfall configurations provide unreliable results, particularly for Krokodil and Benin-Oueme basins in Africa. For MISDc and HYMOD, the overall results in West Africa are even better than in Europe thanks to the strong seasonality of river discharge observations for such basins that allows us to obtain better performance in terms of KGE (due to the high temporal correlation, R > 0.9, that is one of the components of KGE^39^). Among the three rainfall-runoff models, MISDc has been found to perform the best with mean KGE = 0.83 while HYMOD (mean KGE = 0.76) and GR4J (mean KGE = 0.60) show a less good agreement with river discharge observations. Therefore, MISDc model has been selected for the subsequent analyses.

### Rainfall and hydrological assessment in Europe

**Figure 2:**
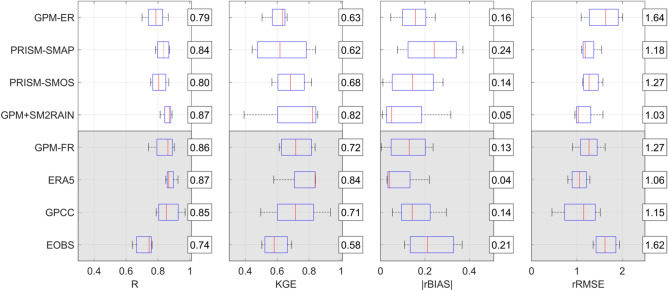
Correlation coefficient, R, Kling-Gupta Efficiency, KGE, absolute value of relative bias, |rBIAS|, and relative root mean square error, rRMSE, for the different products in European basins with respect to reference rainfall. Reference rainfall is PITA for Italian basins and EOBS for Danube basin (Danube basin is not considered for EOBS product). For each boxplot, the horizontal line represents the median values and the box represents the 25th and 75th percentile, the dotted whiskers extend to the extreme data points and cross symbols represent outliers. The numbers in the boxes represent the mean values.

The quality of rainfall products has been firstly assessed in Europe through a comparison with the reference datasets, i.e., PITA (Italian Rainfall Dataset) for the Italian basins and EOBS (ECA&D rainfall dataset) for Danube basin. Results are summarized in Fig. 2 in which the box plots of R (Pearson’s Correlation Coefficient), KGE, absolute value of rBIAS (relative BIAS), |rBIAS|, and rRMSE (relative root mean square error) are illustrated for all products, including EOBS. Long latency products are shown in the grey area to differentiate them from the short latency products. The different scores provide a similar assessment with the products with higher R and KGE also showing lower |rBIAS| and rRMSE. ERA5 is the best long latency products with mean R = 0.87 and KGE = 0.84. ERA5 is also characterized by low |rBIAS| and rRMSE. Among the short latency satellite-only products, GPM+SM2RAIN provides the best agreement with the reference rainfall performing slightly better than GPM-FR and GPCC, on average. In this region, where the density of rain gauge is relatively high and the amount of rainfall information shared by GPCC and the reference rainfall is likely substantial, this result is unexpected. We attributed it to the coarse resolution of GPCC likely not suitable to reproduce the actual rainfall pattern over complex topography regions as in the investigated basins in southern Europe. GPM-ER is performing less good than soil moisture corrected products, particularly in terms of rRMSE, similar to the gauge-based EOBS product. The latter is known to have low accuracy in southern Europe due to the low density of rain gauges used for its development in such area^43^. The results are in good agreement with those reported in^9^ who have shown R values of GPM+SM2RAIN ~ 15 to 20% higher than GPM-ER in Europe.

**Figure 3:**
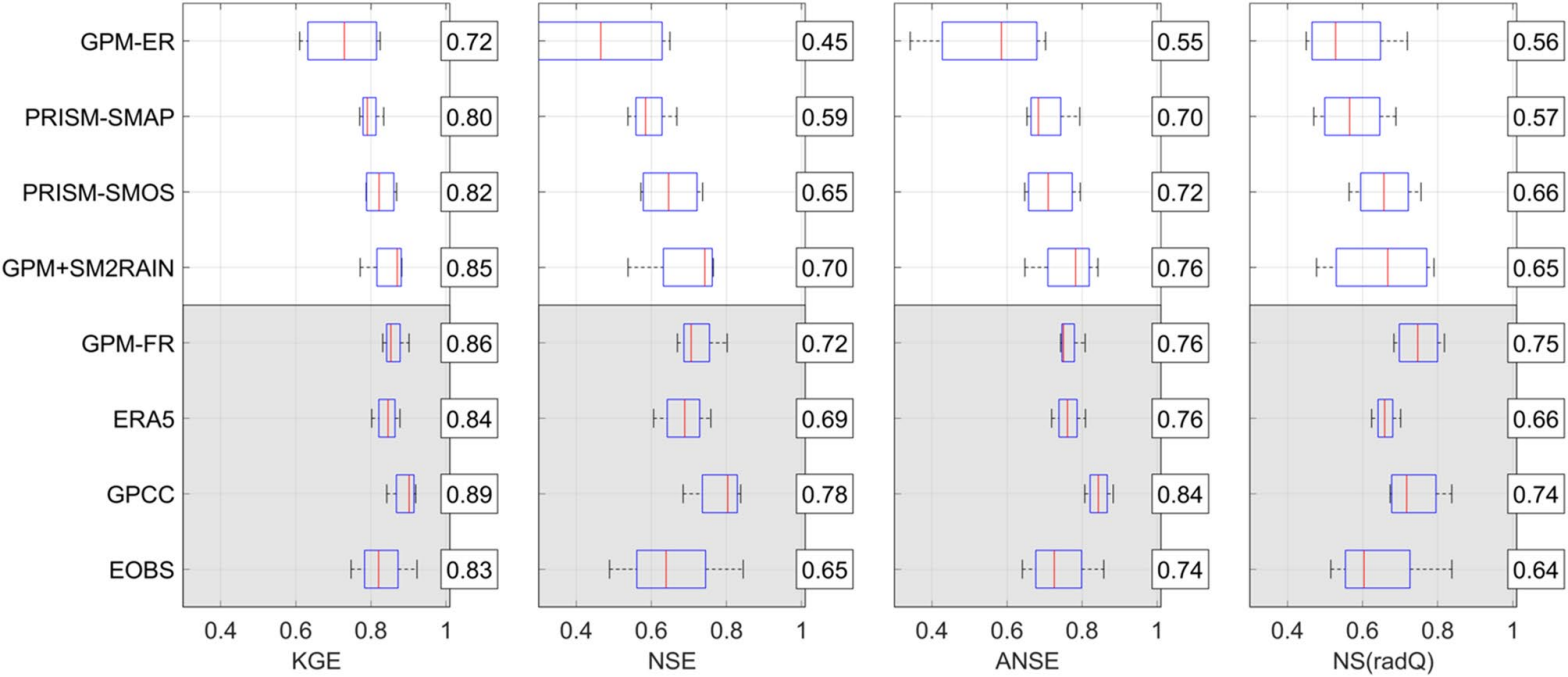
Kling-Gupta Efficiency, KGE, Nash-Sutcliff Efficiency, NSE, NSE for high flows, ANSE, and NSE for low flows, NS(radQ), in the European basins for all products and MISDc model. For each boxplot, the horizontal line represents the median values and the box represents the 25th and 75th percentile, the dotted whiskers extend to the extreme data points and cross symbols represent outliers. The numbers in the boxes represent the mean values. Note that x-axis starts at 0.3 to improve visualization.

Bearing in mind the results of the rainfall assessment, the products performance in terms of river flow prediction in the European basins are analysed. To investigate the variability of MISDc performance for the different products, in Fig. 3, KGE, NSE (Nash Sutcliffe Efficiency), ANSE (NSE for high flows) and NS(radQ) (NSE for low flows) values for all basins separated by product are shown. In terms of KGE, good performances have been obtained with rain gauge observations from GPCC, ERA5 and GPM-FR with a mean KGE equal to 0.89, 0.84 and 0.86, respectively. Differently, EOBS dataset is performing less good (mean KGE = 0.83). Among the four satellite-only rainfall products, GPM+SM2RAIN provides the best scores (mean KGE = 0.85) followed by PRISM-SMOS (mean KGE = 0.82) and PRISM-SMAP (mean KGE = 0.80). GPM-ER provides lower performance (mean KGE = 0.72). For the other performance scores, a similar picture is obtained with GPCC being the best among the long latency products and GPM+SM2RAIN among the short latency products [except for NS(radQ) in which PRISM-SMOS is better on average].

**Figure 4:**
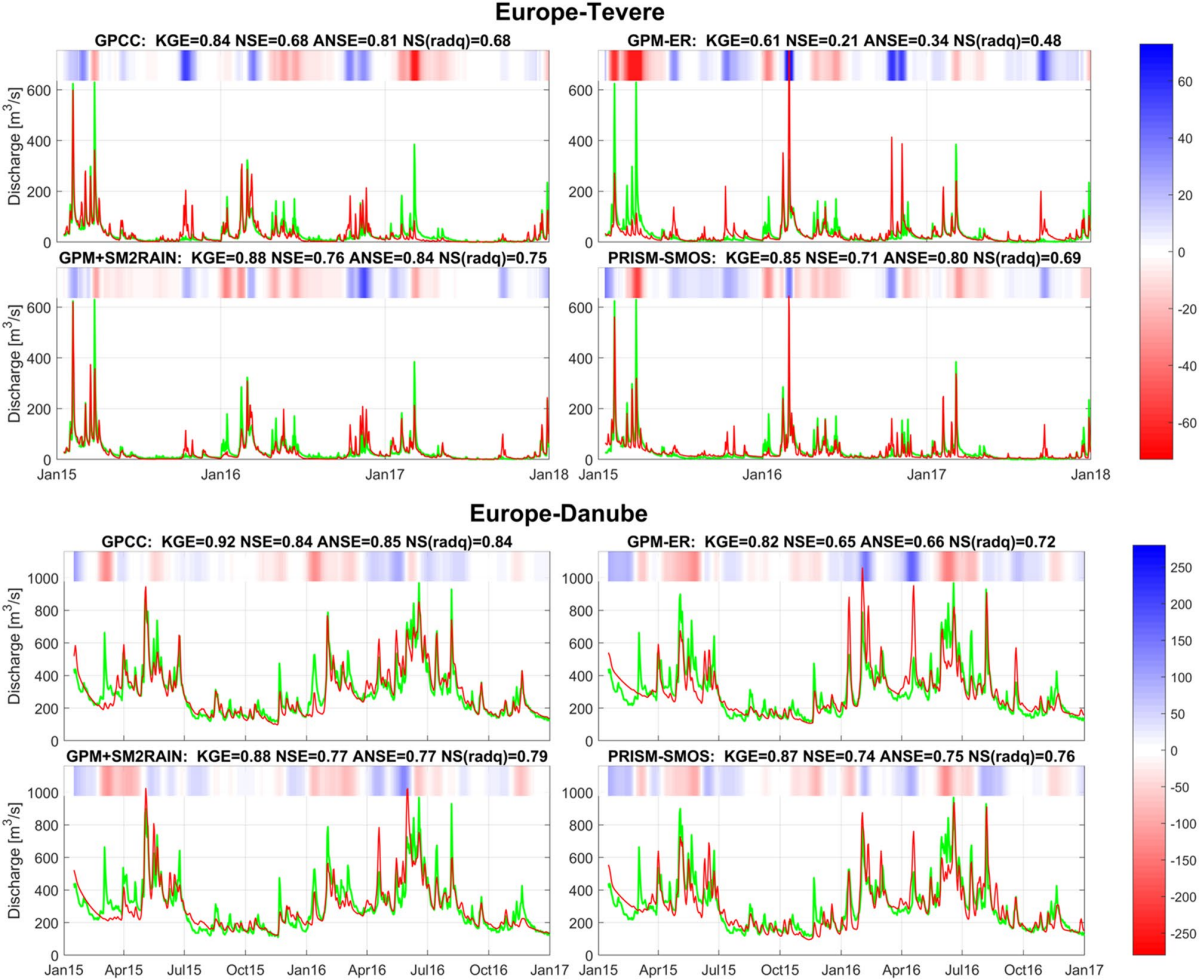
Observed (green tick lines) versus simulated (red lines) river discharge obtained through MISDc rainfall-runoff model for Tevere (top panels) and Danube (lower panels) basin by using as rainfall input: GPCC (top left of each panel), GPM-ER (top right), GPM+SM2RAIN (bottom left), and PRISM-SMOS (bottom right). In the title the performance scores in terms of Kling-Gupta Efficiency, KGE, Nash-Sutcliff Efficiency, NSE, NSE for high flows, ANSE, and NSE for low flows, NS(radQ), is given. In the top of each panel, the coloured bar has darker blue (red) colours for larger positive (negative) errors, i.e., the difference (in m^3^/s) between simulated and observed discharge (see the colorbar on the right), and it helps visualizing the differences between observed and simulated discharge.

Two examples of simulation of river discharge for two basins in Europe (Tevere and Danube, Fig. 4) are shown. For each figure, the simulation with MISDc rainfall-runoff model by using as rainfall input GPCC, GPM-ER, GPM+SM2RAIN and PRISM-SMOS is illustrated. In the title of each subplot the performance scores are shown in terms of KGE, NSE, ANSE, and NS(radQ). KGE is considered as the most important scores for river discharge simulation and the rainfall-runoff models are calibrated by maximizing this score. The overall results highlight the good performance of GPM+SM2RAIN and PRISM-SMOS (slightly better than PRISM-SMAP). Moreover, the performances of GPM+SM2RAIN and PRISM-SMOS are always better than GPM-ER indicating that the integration of soil moisture is highly beneficial, both for the reproduction of medium (as assessed by KGE) and particularly of high (as assessed by ANSE) flows. A good example is visible for the Tevere basin (Fig. 4, top panels) where the integrated products (GPM+SM2RAIN and PRISM-SMOS) corrects the overestimation of GPM-ER at the beginning of 2016 and the underestimation at the beginning of 2015.

### Triple collocation analysis

For each basin (including the European basins), the mean areal rainfall from the seven rainfall products has been computed and used in the Triple Collocation analysis. Table 1 summarizes the results for all the products, except GPM-FR, and for all the basins. In the table, it is highlighted in bold the best product for each basin and in italic the second-best product. For West Africa basins, the best product is GPM+SM2RAIN and PRISM-SMAP is the second best. This result clearly shows that for these basins, in which the density of rain gauge is low, the satellite products could be a valid alternative to both gauge-based and reanalysis rainfall products. In South Africa basins, GPCC is the best product and GPM+SM2RAIN is the second best. In Europe, all the products perform better that in Africa basins with GPCC, ERA5 and GPM+SM2RAIN among the best performing products. The latter results are in good agreement with the classical validation performed in the previous section. Based on the overall results, on average GPM+SM2RAIN has the higher Triple Collocation correlation (average TC-R = 0.91) followed by GPCC (0.84), ERA5 (0.83), and PRISM-SMAP (0.821). By comparing the performance of the soil moisture corrected products (GPM+SM2RAIN, PRISM-SMOS, and PRISM-SMAP) with GPM-ER, we have found that they always outperform GPM-ER suggesting a significant benefit of the integration of satellite-based soil moisture. Specifically, the improvement is equal to 7% and 5% for PRISM-SMAP and PRISM-SMOS, respectively, and it reaches 18% for GPM+SM2RAIN. Again, results are in accordance with previous studies (see e.g., Fig. 9 in^9^) showing good performance of GPCC in South Africa and low performance of both GPCC and ERA5 in West Africa. GPM+SM2RAIN is performing good throughout Africa except over dense forests and desert areas in which the error of satellite soil moisture observations is high and the use of such products is discouraged or masked out.

**Table 1:** Triple Collocation (TC) performance, in terms of TC correlation coefficient, TC-R, for the different satellite rainfall products investigated in this study.

	Europe				West Africa			South Africa			Average
	Tevere	Po	Ronco	Danube	Benin-Oueme	Daka-Saidou	Gourbassy	Krokodil	Tsitsa	Tsomo	
GPCC	*0.898*	0.917	**0.930**	0.911	0.732	0.576	0.725	**0.920**	**0.919**	**0.911**	*0.844*
ERA5	0.878	*0.930*	*0.888*	*0.913*	0.725	0.783	0.698	0.795	0.839	0.856	0.831
GPM+SM2RAIN	**0.921**	**0.940**	0.882	**0.931**	**0.955**	**0.923**	**0.938**	*0.877*	*0.858*	*0.874*	**0.910**
PRISM-SMOS	0.827	0.888	0.787	0.853	0.803	0.766	0.769	0.822	0.763	0.764	0.804
PRISM-SMAP	0.873	0.913	0.817	0.868	*0.807*	*0.806*	*0.799*	0.846	0.726	0.756	0.821
GPM-ER	0.793	0.902	0.718	0.824	0.762	0.748	0.789	0.748	0.689	0.707	0.768
Average	0.865	0.915	0.837	0.883	0.797	0.767	0.786	0.835	0.799	0.811	

### River flow and evapotranspiration prediction in Africa

**Figure 5:**
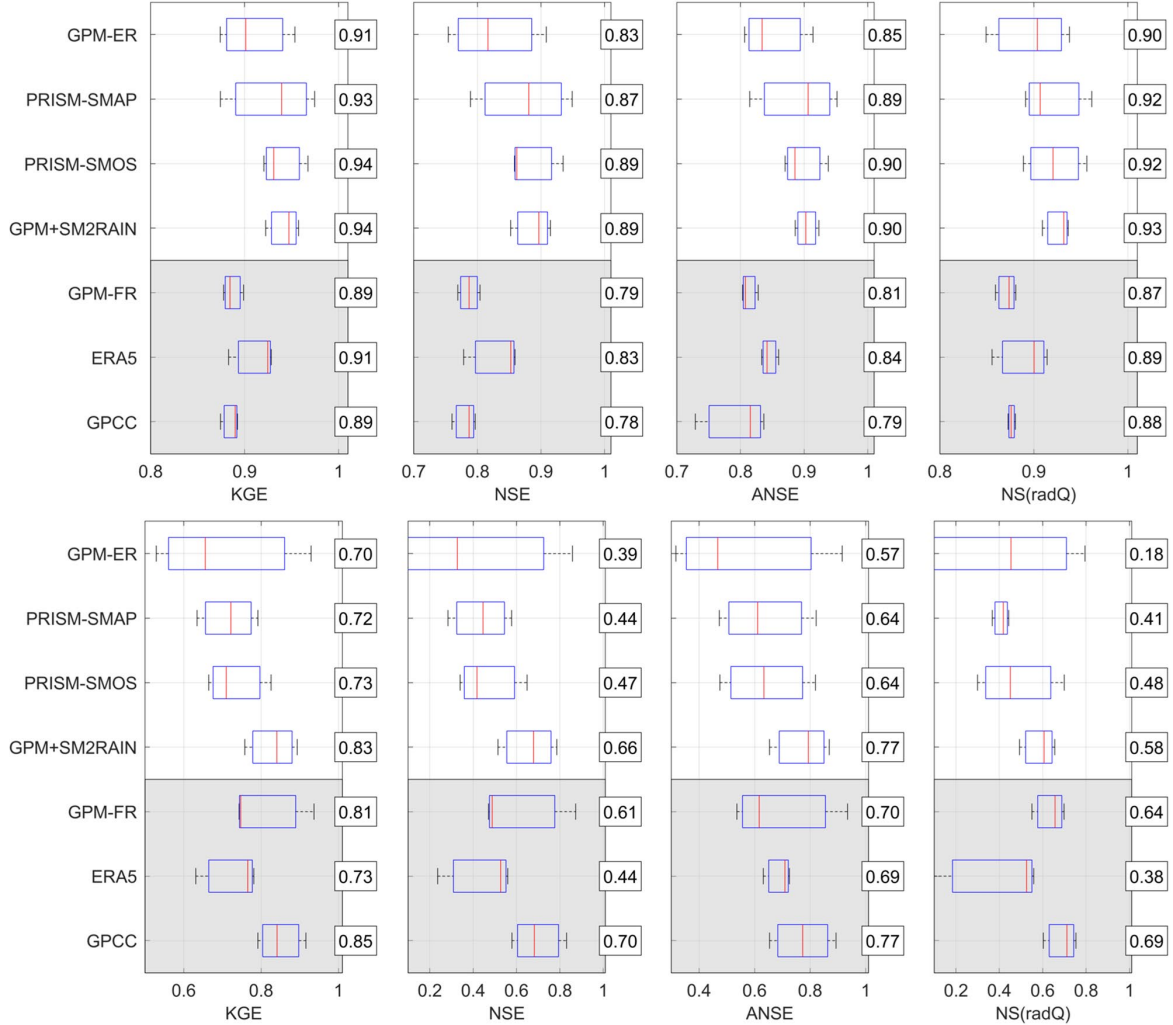
As in Fig. 3 for West Africa (top panels) and South Africa (bottom panels). Note that x-axis starts at different values to improve visualization.

Figure 5 shows KGE, NSE, ANSE and NS(radQ) performance scores grouped for the West Africa (top panels) and South Africa (bottom panels). Some interesting points can be summarized by analysing Fig. 5:

- Gauge-based\corrected and reanalysis products (GPCC, ERA5, GPM-FR) perform well in South Africa, as expected, due to the presence of rain gauges, whereas in West Africa, they perform worse than soil moisture-corrected products (GPM+SM2RAIN, PRISM-SMOS, and PRISM-SMAP) and equal to GPM-ER, on average;
- GPCC (ERA5) performs best among the long latency products in South Africa (West Africa);
- GPM-FR outperforms GPM-ER in South Africa, but not in West Africa due to the scarcity of rain gauges to correct the product, therefore gauge correction in West Africa should be avoided due to the low density of rain gauges;
- In West Africa, the soil moisture-corrected products (GPM+SM2RAIN, PRISM-SMOS, PRISM-SMAP) perform very good with mean KGE and NSE higher than 0.93 and 0.87, respectively, and better than the long latency products;
- Overall, GPM+SM2RAIN outperforms the PRISM-based products, particularly in South Africa where GPM-ER is performing low (mean KGE = 0.58 and NSE = 0.39);
- GPM+SM2RAIN shows the better scores in terms of high flows (NSE and ANSE).

**Figure 6:**
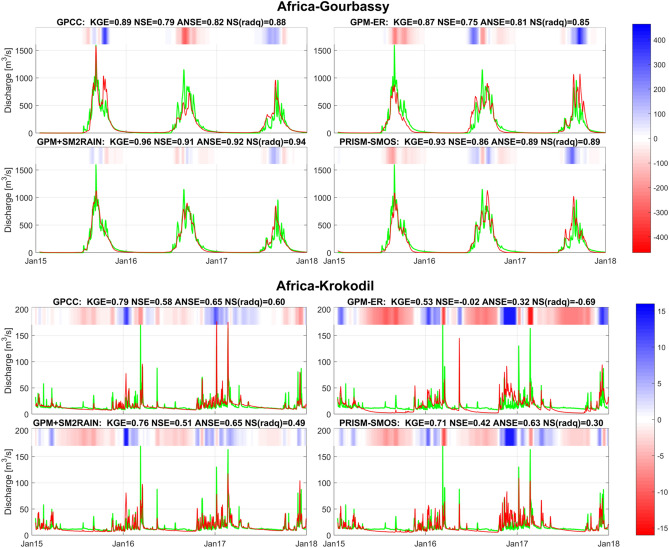
As in Fig. 4 for Gourbassy basin in West Africa (top panels) and Krokodil basin in South Africa (bottom panels).

Figure 6 shows the observed and simulated river discharge for Gourbassy (top panels) and Krokodil (bottom panels) basins by using GPCC, GPM-ER, GPM+SM2RAIN and PRISM-SMOS as input rainfall. In Gourbassy basin all products are able to reproduce observed river discharge satisfactorily and GPM+SM2RAIN is performing the best, even better than GPCC (see also^29^). However, in Krokodil basin, also due to the higher temporal variability of river discharge, the products performance is lower. Indeed, it is evident the problem of GPM-ER in providing reliable simulations (NSE < 0); such problem is largely corrected from the integrated products (NSE > 0.4) particularly during the flood period October 2016–March 2017.

An additional analysis has been carried out for African basins by comparing the simulated actual evapotranspiration data with Global Land Evaporation Amsterdam Model (GLEAM^49,50^) dataset over the six African basins. As the model is calibrated against river discharge observations, this assessment might be considered as an independent validation that also ensures the consistent closure of the water balance. Indeed, we expect that higher quality rainfall products provide a better agreement with evapotranspiration fluxes provided by GLEAM. Results have been evaluated in terms of R to assess products capability to reproduce the temporal variability of actual evapotranspiration. For all basins, performances are relatively good with R values ranging between 0.7 and 0.85. For all African basins, GPM+SM2RAIN is slightly outperforming the others with mean R equal to 0.79, followed by PRISM-SMOS, GPCC and ERA5 (0.78). In West Africa the best score is obtained from GPM-ER (0.82) while in South Africa from GPM+SM2RAIN (0.81). The good performance of the integrated products (GPM+SM2RAIN and PRISM-SMOS) might be attributed to the use of satellite soil moisture data that assure a better closure of the water balance and thus provide better evapotranspiration estimates. However, such preliminary results have to be confirmed from the analysis over a larger number of basins (and longer time periods).

In order to investigate the reasons for the different performance of GPM+SM2RAIN and PRISM-based products, we have computed the daily correlation of these rainfall products with GPCC and GPM-ER. The correlation of PRISM-based products and GPM-ER is higher than 0.93 thus highlighting that the soil moisture correction with PRISM provides a rainfall product similar to GPM-ER. Differently, the correlation of GPM+SM2RAIN and GPM-ER is lower and, on average, equal to 0.82. Therefore, in regions where GPM-ER is not performing well (e.g., South Africa), the lower performance of PRISM-based products with respect to GPM+SM2RAIN might be attributed to their stronger dependence with GPM-ER.

## Discussion and conclusions

The hydrological validation of satellite rainfall products has several advantages and benefits with respect to the validation with ground rainfall observations:

1. The assessment is carried out with river discharge observations and hence it is fully independent from ground rainfall observations that are used in gauge-corrected products. Therefore, the problem of validating products including ground observations (e.g., GPM-FR) is overcome.
2. The assessment is carried out at basin scale. Therefore, for basins larger than 1,000 km^2^ the scale of the validation data is comparable with the resolution of the satellite products thus solving the point to area validation problem encountered when rain gauge observations are used (e.g.,^40^).
3. The results provide direct information on the use of the satellite rainfall product for hydrological applications, e.g., river flow prediction and water resources management. Therefore, the real-world impact of the products is assessed.

In the hydrological validation, the latency is fundamental. Indeed, products with latency larger than 3 days have very little/no use in operational hydrological applications. In this study, we have performed the hydrological validation also for long latency products (GPCC, ERA5, and GPM-FR), but their actual use for hydrological applications is not feasible.

The results of the classical rainfall validation (Fig. 2), Triple Collocation (Table 1) and of the hydrological validation of the satellite rainfall products (Figs. 3,5) are consistent. Specifically, it is evident that in the three analyses, the soil moisture corrected products outperform the GPM-ER dataset, and, on average, the best performing product is GPM+SM2RAIN. This consistency adds value to the overall analysis performed in this study that can be considered robust and reliable. However, it should be underlined that in regions characterized by frozen soils, snow and dense forests, satellite soil moisture products accuracy is expected to be low and hence the correction will be not effective.

A potential reason for the better performance of the GPM+SM2RAIN product for high flows should be attributed to the correction with satellite soil moisture data. Indeed, GPM-ER data are based on instantaneous snap shots a few times a day, that may miss critical storms between the satellite over passes. Rain gauge datasets may miss small storms between the stations. Consequently, the simulated river flow based on GPM-ER or rain gauge rainfall data only may be underestimated, especially when there are many or heavy missed storms that cause high flows. Satellite soil moisture data may catch the footprints of the missed storms and thus may help improve the river discharge forecasts if they are used to correct the rainfall data.

Four limitations of this study can be identified. Firstly, the use of a short time period (2–3 years) for performance assessment might be too short to obtain reliable results. We have partly addressed this problem by using three different rainfall-runoff models and without splitting the period in calibration and validation. However, future studies will be performed with a longer time period thanks to the recent availability of GPM products reprocessed starting in 2000. Secondly, the use of lumped rainfall-runoff models for performing hydrological predictions over large basins might be questionable. The overall good performances that we have obtained (see Fig. 1) allows us to conclude that the results are robust but the use of distributed rainfall-runoff modelling is foreseen for future studies (e.g.,^23^). Thirdly, the number of basins in which the assessment has been carried out is limited to ten as the collection of recent data of river discharge, particularly over African basins, is particularly challenging. As above, the use of a larger number of basins is among the next step for this research activity. Fourthly, the rainfall-runoff models have been calibrated in each basin and the obtained performance might be partly influenced by the tuning of the parameters. The testing of the products without calibration will be the object of future investigations once a larger number of basins and longer time series will be available.

It should be highlighted that, to our knowledge, this is the first study performing the hydrological validation of GPM products in Africa. In addition, the validation of three long latency products has been carried out to answer the research question: do the latest satellite rainfall products outperform ground-based datasets for river flow prediction? The main outcome of this study is the significant benefit of integrating satellite soil moisture data for improving the performance of satellite rainfall products for hydrological simulations^25,26^. The improvement of soil moisture-corrected rainfall products with respect to GPM-ER is around 20% in terms of KGE for GPM+SM2RAIN (from 0.74 to 0.87, Fig. 2); and slightly lower for PRISM-based products (0.85 for PRISM-SMOS and from 0.74 to 0.82 for PRISM-SMAP). Differently from previous studies (see e.g.,^1,23,30,32^, we have found that GPM+SM2RAIN is outperforming the long latency products based on rain gauge and reanalysis. This result should be attributed to: (1) the high-quality of the GPM+SM2RAIN rainfall product which is based on the latest GPM satellite product and integrated with soil moisture derived rainfall from multiple satellites/sensors (SMOS, SMAP and ASCAT), and (2) the low density of rain gauges particularly in West Africa basins. The assessment in terms of reproduction of actual evapotranspiration has confirmed the slightly higher quality of GPM+SM2RAIN with respect to gauge-based and reanalysis products over African basins.

It should be underlined that GPM+SM2RAIN and PRISM-based satellite rainfall products are potentially available in with a latency < 3 days, thus representing an important new data source for river flow forecasting worldwide. The good results of the hydrological validation of these newly derived short latency satellite rainfall products highlight the strong need to disseminate such products for hydrological (and agricultural) applications within developing countries (e.g., Africa and South America).

## Methods

### Study area and datasets

**Figure 7:**
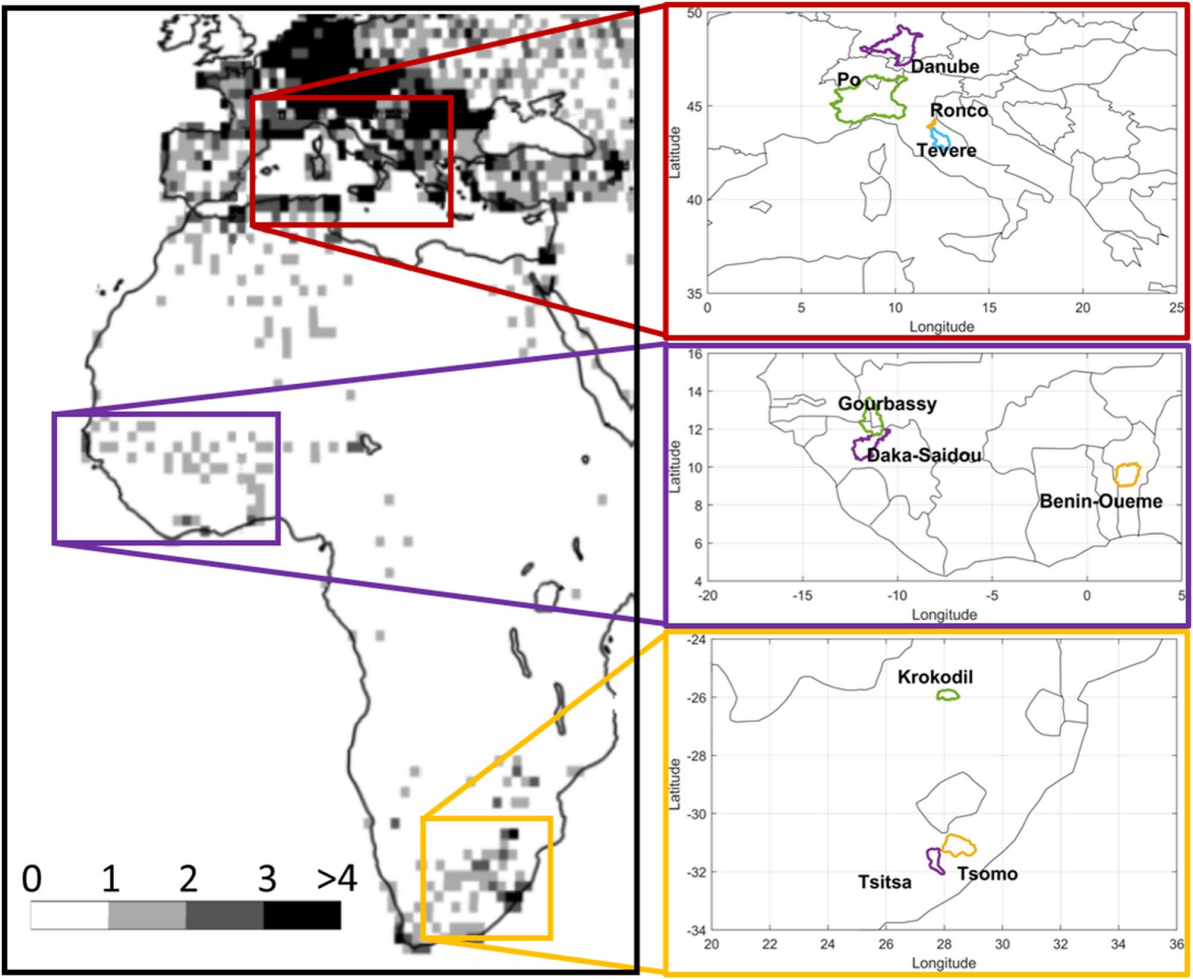
Location of the study basins selected for the hydrological validation in Europe (top right), West Africa (middle right), and South Africa (bottom right). On the left the number of stations used for the GPCC First Guess 1.0° product for years 2015–2018 are shown. The map has been generated by using Matlab software.

To perform a robust assessment of the satellite rainfall products for river flow prediction, a dataset of six basins in Africa and four basins in Europe has been collected (see Fig. 7; Table 2). The selection of the basins has been primarily driven by river discharge data availability in the recent period 2015–2017, which are quite rare, particularly for basins in Africa. River discharge data in West and South Africa has been collected through direct contact with local river basins authorities from the authors, and hence a limited number of 6 basins has been made available at the time of writing. Basin area ranges from 548 km^2^ for Ronco basin to 55,183 km^2^ for Po basin (at Boretto station) thus covering a wide range of basin sizes. For each basin, daily river discharge observations for the period 2015–2017 are available; for some basins, data are available only from 2015 to 2016. The density of rain gauges in each basin, as obtained from GPCC dataset, is also reported in Table 2. Air temperature data have been collected from the National Centers for Environmental Prediction (NCEP) reanalysis dataset^41^ and they are used for computing mean areal evapotranspiration at the basin scale through the adapted Blaney and Criddle Equation^42^.

**Table 2:** List of the selected basins with the information on the location, the basin area, the density of rain gauges (from GPCC) and the data period.

Basin name	Station	Region	Area (km^2^)	#rain gauges/10,000 km^2^ (GPCC)	Data period
Po	Boretto	Europe	55,183	> 4	2015–2016
Tevere	Monte Molino	Europe	5,269	> 4	2015–2017
Ronco	Coccolia	Europe	548	> 4	2015–2016
Danube	Ingolstadt	Europe	20,001	> 4	2015–2016
Benin-Oueme	Beteorou	West Africa	12,000	1–2	2015–2016
Daka-Saidou	Sabere Bani	West Africa	15,659	1	2015–2017
Gourbassy	Diokéba	West Africa	16,134	1	2015–2017
Krokodil	Kalkheuwel	South Africa	2,551	2–3	2015–2017
Tsitsa	Xonkonxa	South Africa	4,285	2–3	2015–2017
Tsomo	Wyk Maduma	South Africa	2,359	2–3	2015–2017

### Precipitation datasets

Daily precipitation observations have been collected from nine different datasets.

Two reference datasets have been collected in Europe. Firstly, the ECA&D rainfall dataset E-OBS (EOBS) gridded dataset has been considered^43^. The station dataset comprises a network of 2,316 stations, with the highest station density in Northern and Central Europe and lower density in the Mediterranean. We have used the 0.25° regular latitude–longitude grid with daily resolution. Secondly, in Italy, gridded rainfall data provided by 3,000+ stations of the National Department of Civil Protection^20^ have been used (PITA). The data are gridded at 0.1° resolution and aggregated at daily temporal scale.

Ground-based observations are collected from GPCC^38^ that is a global dataset at 1° resolution and daily time step. The gauge density of GPCC is lower than EOBS and particularly PITA, therefore GPCC is used not as reference but only as alternative gauge-based product. Reanalysis is taken from the latest ECMWF reanalysis ERA5^37^ with a temporal resolution of 1-h and spatial resolution of nearly 36 km. As gauge-corrected satellite product, we have used the Integrated Multi-Satellite Retrievals for GPM (IMERG) Final Run version 5^11^ for which ground observations from GPCC are used to correct monthly accumulation successively rescaled at hourly time scale. IMERG Final Run, hereinafter GPM-FR, is available from 2014 at 0.1° spatial resolution and with a temporal resolution of 30-min. These three products, i.e., GPCC, ERA5 and GPM-FR are available with a latency of 1+ months.

Four different satellite-only products have been used. The IMERG Early Run version 5, hereinafter GPM-ER^11^ is characterized by a spatial\temporal resolution of 0.1°\30-min. Three products that integrates the top down GPM-ER with satellite soil moisture data have been tested. The SM2RAIN-based satellite rainfall product, i.e., GPM+SM2RAIN^9^, integrates GPM-ER with SM2RAIN-based product applied to SMAP, SMOS and ASCAT satellite soil moisture. GPM+SM2RAIN (https://zenodo.org/record/3345323) has a spatial resolution of 0.25° and daily temporal resolution. Two PRISM-based satellite rainfall products have been considered^15,36^. The PRISM method is applied to correct GPM-ER with SMOS (PRISM-SMOS) and SMAP (PRISM-SMAP) soil moisture observations. PRISM-SMOS and PRISM-SMAP have a spatial resolution of 0.25° and 3-h temporal resolution. Details on the characteristic of GPM+SM2RAIN algorithm and on PRISM approach is given in^11,36^ and we refer the interested reader to these publications for more specific information. Here, briefly, we underline the main difference between the two approaches that relies on the method used for integrating soil moisture observations. In GPM+SM2RAIN, a soil moisture-based rainfall product obtained from the application of SM2RAIN to satellite soil moisture products (SMAP, SMOS and ASCAT) is merged with GPM-ER via the Optimal Linear Combination approach^51^. In PRISM, satellite soil moisture data (SMOS and SMAP) are assimilated into a simplified soil moisture model using GPM-ER as input. The innovations after the assimilation are exploited for correcting the input precipitation data thus obtaining the PRISM-based products.

A summary of the precipitation products used in this study is given in Table 3. For each basin, the precipitation data have been aggregated at basin scale spatially averaging the observations of all cells contained in the basin and resampled at daily temporal resolution for the products available at sub-daily time scale (i.e., GPM-ER, GPM-FR, and ERA5).

**Table 3:** List of the selected rainfall products for this study.

Product	Data period	Spatial resolution (°)	Temporal resolution	Latency	References
**Reference products**					
EOBS	1950-on going	0.25	1-day	3 months	^46^
PITA	1978-on going	~ 0.1	1-day	15 days	^34^
**Long latency products (> 1 month)**					
GPCC	1950-on going	1	1-day	15–45 days	^35^
ERA5	1978-on going	0.36	1-h	3 months	^34^
GPM-final run	2014-on going	0.1	30-min	> 1 month	^9^
**Short latency products (< 3 days)**					
GPM-early run	2015-on going	0.1	30-min	12 h	^9^
GPM+SM2RAIN	2015-on going	0.25	1-day	3 days	^7^
PRISM-SMOS	2015-on going	0.25	3-h	3 days	^13,33^
PRISM-SMAP	2015-on going	0.25	3-h	3 days	^13,33^

For each basin, and for each rainfall-runoff model (see “Discussion and conclusions”), seven river discharge simulations have been carried out, i.e., by using as precipitation input three long latency (> 1 month) products: (1) GPCC, (2) ERA5, and (3) GPM-FR; and 4 short latency (< 3 days) products: (1) GPM-ER, (2) GPM+SM2RAIN, (3) PRISM-SMOS, and (4) PRISM-SMAP.

### Actual evaporation dataset

In African basins, the assessment of products performance has been carried out also considering the capability of the rainfall-runoff model to reproduce the temporal variability of actual evapotranspiration, another important component of the water balance. As reference, we have considered the dataset obtained from the Global Land Evaporation Amsterdam Model (GLEAM^49,50^). Specifically, the actual evapotranspiration dataset from GLEAM v3.3b has been considered, i.e., not including ERA5 data as input, which has a spatial resolution of 0.25°, daily temporal resolution and covers the period 2003–2018. As for the precipitation dataset, actual evapotranspiration data have been aggregated at basin scale spatially averaging the observations of all cells contained in the basin.

## Rainfall-runoff models

### MISDc

MISDc—“Modello Idrologico Semi-Distribuito in continuo”—is a continuous rainfall-runoff model developed by Brocca et al.^44^ for the operational forecasting of flood events in central Italy. In this study, a two-layer version of the model has been used. With respect to the previous version, it includes a snow module and a different infiltration equation. The model uses as input daily rainfall and potential evapotranspiration data and simulates the temporal evolution of river discharge, actual evapotranspiration and soil moisture for a surface and a root-zone soil layer. Water is extracted from the first layer by evapotranspiration, which is calculated by a linear function between the potential evaporation and soil moisture. A non-linear relation is used for computing the percolation from the surface to the root-zone layer. The rainfall excess is calculated by a power law relationship as a function of the first layer soil moisture while base flow is a non-linear function of the soil moisture of the second layer. The MISDc version used for this study has nine parameters to be estimated by calibration against ground-based river discharge observations. Full details on model equations are given in^44^ and recent applications with satellite observations can be found in^1,26^.

### GR4J

GR4J is a lumped bucket-type model that represents the rainfall-runoff relationship using an interception function, two stores, a unit hydrograph and an exchange function^45^. GR4J operates at a catchment scale with a daily time-step. The development of the GR4J model was initiated by Claude Michel at the beginning of the 1980s at Cemagref. The first version of the model only had a single parameter. Further development of the GR4J model was undertaken using a modelling approach where large numbers of catchments were used to evaluate and improve the model. The GR4J version used for this study has five parameters to be estimated by calibration against ground-based river discharge observations and uses as input daily rainfall and potential evapotranspiration data.

### HYMOD

HYMOD is a step rainfall excess model based on a nonlinear water storage capacity distribution function^46^. The routing system includes a sequence of three quick-flow tanks which describe surface flow, in parallel to a slow-flow tank for groundwater. The HYMOD model is a flexible solution that is increasingly adopted for its capability of providing a good fit in several practical applications. It assumes that each point location in the basin is characterised by a local value of soil water storage, which varies from zero in the impervious areas up to a maximum value in the most permeable location of the catchment. Soil water storage is assumed to be randomly varying, so that for an assigned value of soil water storage a probability distribution is introduced. The HYMOD version used for this study has five parameters to be estimated by calibration against ground-based river discharge observations and uses as input daily rainfall and potential evapotranspiration data.

### Processing steps for performing the hydrological validation

The use of satellite-based rainfall products for river flow modelling requires some pre-processing steps. We have employed here the same approach as in Camici et al.^1^ in which the rainfall-runoff model has been recalibrated for each of the different rainfall product by maximizing the Kling–Gupta efficiency (KGE^39^) with respect to observed daily discharge. KGE is a performance index with optimal value equal to 1; good, satisfactory and poor performance is obtained for KGE between 0.7 and 1, 0.4 and 0.7, lower than 0.4, respectively. In addition to KGE, the Nash–Sutcliffe efficiency, NSE^47^, the NSE for high flows, ANSE^1^, and the NSE for low flows, NS(radQ), i.e., computed on the squared root of river discharge, performance scores have been employed. For all these scores the optimal value is equal to 1 and negative values mean poor performances. NSE, ANSE are to be used for assessing model performances for high and extremely high flows, respectively, while NS(radQ) for low flow conditions. Therefore, for each rainfall product and basin, the parameter values of the three rainfall-runoff models have been calibrated in a physically reasonable range of values to maximize KGE. A validation period has not been considered in this analysis due to the limited length of the data period. Certainly, this is a limitation of the study as it does not allow for the evaluation of the satellite rainfall product in an operational context. However, here we have aimed to assess the best information that can be extracted from each rainfall product by using the maximum length of the available data (limited to 3 years). Moreover, as all the products have been treated with the same procedure, the fair comparison between them is ensured.

### Rainfall assessment in Europe

In Europe, the assessment of rainfall products has been carried by considering classical statistical scores, i.e., the correlation coefficient, R, KGE, the relative bias, rBIAS, i.e., the bias normalized with the temporal mean of the observations, and the relative root mean square error, rRMSE, i.e., the RMSE normalized with the temporal mean of the observations. As reference, we have used the PITA dataset (based on 3,000+ rain gauges) for Italian basins and EOBS for Danube basin (the quality of EOBS is found not very good in Italy).

### Triple collocation analysis

To assess the quality of rainfall products in Africa, we have performed the Triple Collocation analysis as in Massari et al.^6^. Indeed, for the African basins, we do not have a rainfall product to be considered as reference, and Triple Collocation allows us to obtain an estimation of the error variances of each product without the need of a reference. An additive error model has been assumed, and the extended Triple Collocation approach^48^ has been applied thus providing the correlation against the hypothetical truth of three independent rainfall products^9,18^, hereinafter TC-R. The additive error model has been selected as it has been found better than the multiplicative model^6^ if daily rainfall observations are analysed. Daily rainfall observations are characterised by a large number of zeroes, particularly over dry areas, that deteriorate the accuracy of Triple Collocation if a multiplicative error model is assumed. For details on the implementation of Triple Collocation, the reader is referred to Massari et al.^6,9^. In the selection of the triplets, the independence of the errors of the rainfall products has to be ensured. For instance, the two GPM products cannot be included in the same triplet, and similarly GPCC and GPM-FR that uses GPCC observations for correcting monthly accumulations. Therefore, for the assessment of the products the triplets have been formed by using GPCC, ERA5 and the four satellite-only products (GPM-ER, GPM+SM2RAIN, PRISM-SMOS, and PRISM-SMAP). GPM-FR, including both GPCC and GPM-ER, has not been assessed in this analysis as the independence with the other products cannot be ensured.
